# A Case of Hepatic Stone, &c.

**Published:** 1889-04

**Authors:** E. T. Saunders

**Affiliations:** Huntington, W. Va.


### A CASE OF HEPATIC STONE SUCCESSFULLY TREATED.

BY E. T. SAUNDERS, M. D., HUNTINGTON, W. VA.

In June, 1888, I was summoned to attend Benj. D. æt. 52 years, residing in the county about twenty-two miles from this point, and on arrival found him suffering with severe cramps about the situation of the common choledoc duct, radiating along the intestinal tract. From the history of the case I could ascertain but little, except that for several years he had been thus suffering at intervals, and had been under the supervision of several physicians, though having failed to obtain either a permanent cure, or even relief from pain during the exacerbations. Before having looked into the case, I immediately administered ¼ gr. of morphine hypodermically, and in conjunction with fomentations locally applied, used a foot bath. From this treatment the patient obtained signal relief; the pain, however, returning about 10 o'clock that night, when I again repeated the morphine hypodermically. The following morning I prescribed some simple medicine intended as a solvent, gave general directions and returned home.

After the lapse of a few days, I was again summoned to visit my patient suffering as above and during that intermission, upon calm reflection, arrived at a conclusive diagnosis as above. On my arrival I found him in about the same condition as heretofore. I then informed Mr. D. that he was evidently suffering from gall-stone, and I directed a half pint of sweet oil to be taken at once, to be repeated in three hours and as soon as there was an alvine discharge, to wash out the fæces carefully and the stone would be obtained. My object in giving the oil and in such commanding doses was two-fold: First, by the nausea produced to relax the system and thereby favor the passage of stone through the hepatic duct; and secondly, to act as a purgative, thus eliminating them.

Having obtained the desired effect, the fæces were washed out and quite a large quantity of biliary calculi were obtained. This treatment, in conjunction with other minor measures, I continued for several weeks, with complete relief to the patient, obtaining stone after each administration of the sweet oil until there ceased to be a formation, and expulsion of calculi. Having obtained a specimen of these calculi from time to time, I subjected them to a thorough chemical analysis, ascertaining them to be of an acid reaction, rather contrary to my anticipation, and concluded treatment by placing the patient on an alkaline agent that the further formation might thus be prevented by neutralization. After this, all symptoms and exacerbations disappeared, Mr. D. having gained his strength and flesh and is now cured, and able to attend to domestic and other duties. I must not fail to mention the oil plan, together with other accessory treatment, upon general principles, was continued for nearly three months before a positive cure became apparent. I have related, in outline, the above case to many of my professional friends, together with the heroic treatment by sweet oil in such commanding and sickening doses, and find it new to their experience, both as regards the use of that particular medicine and also as to quantity.

Hoping this imperfect epitome of the case may prove of interest and help to the profession in the alleviation and cure of such lesions, and induce others to record some of their observations, I have published the above. [The sweet oil treatment is not new as Dr. S. states, but a number of cases treated by this method may be found reported in the Journals during the last ten years.—ED. SOU. MED. REC.]

